# Potential of histone deacetylase inhibitors for the therapy of ovarian cancer

**DOI:** 10.3389/fonc.2022.1057186

**Published:** 2022-11-25

**Authors:** Fengyi Guo, Hongjing Wang

**Affiliations:** ^1^ Department of Gynecology and Obstetrics, West China Second University Hospital, Sichuan University, Chengdu, Sichuan, China; ^2^ Key Laboratory of Birth Defects and Related Diseases of Women and Children (Sichuan University), Ministry of Education, Chengdu, Sichuan, China

**Keywords:** ovarian cancer, epigenetics, histone acetylation, histone deacetylase, histone deacetylase inhibitor

## Abstract

Malignant ovarian tumors bear the highest mortality rate among all gynecological cancers. Both late tumor diagnosis and tolerance to available chemotherapy increase patient mortality. Accumulating evidence demonstrates that histone modifications play a key role in cancerization and progression. Histone deacetylases is associated with chromatin condensed structure and transcriptional repression and play a role in chromatin remodeling and epigenetics. Histone deacetylases are promising targets for therapeutic interventions intended to reverse aberrant epigenetic associated with cancer. Therefore, histone deacetylases inhibitors could be used as anti-cancer drugs. Preclinical studies have shown promising outcomes of histone deacetylases inhibitors in ovarian cancer while clinical trials have had mixed results and limited success as monotherapy. Therefore, combination therapy with different anticancer drugs for synergistic effects and newly selective histone deacetylases inhibitors development for lower toxicity are hot issues now. In this review, we summarize the latest studies on the classification and mechanisms of action of histone deacetylase and the clinical application of their inhibitors as monotherapy or combination therapy in ovarian cancer.

## Introduction

It is estimated that 21,410 new cases of ovarian cancer (OC) are diagnosed and 13,770 cases of deaths occur due to OC in the United States in 2021 (1). Due to the lack of typical clinical symptoms or only non-specific symptoms in the early stage of OC, more than 75% of cases have progressed to the advanced stage at the time of diagnosis, contributing to a five-year survival rate of only 30% (2). Standard treatment consists of platinum and paclitaxel chemotherapy followed by cytoreductive surgery (CRS) in clinical. Patients usually tolerate treatment well and go into remission, but disease relapse is common (3). Epigenetics refers to the heritable phenotypic changes in genes without changes in nucleotide sequence, and the role of epigenetics in tumor formation is increasingly recognized (4). In this review, we summarize the latest studies on the classification and mechanisms of action of HDACs and the clinical application of their inhibitors in ovarian cancer.

Epithelial ovarian cancer (EOC) is the largest subgroup of ovarian cancer, accounting for around 90%. It includes serous, endometrioid, clear cell, mucinous, and mixed carcinomas (3). Approximately 60–80% of EOC patients will undergo complete remission after surgery combined with chemotherapy, but approximately 80% of these patients have chemotherapy-resistant relapses (5). Even in patients with late relapses who are fully on second-line therapy, the time to second remission is shorter than the time to first remission in more than 95% of cases (6).

Cancers arise from genetic alterations, however, the role of epigenetics in tumor formation is now increasingly recognized (4). EOC occurs as a result of the accumulation of genetic mutations and epigenetic changes leading to malicious transformation of epithelial cells, stem cells, or transient metaplastic regions at the primary site (ovary or fallopian tube) (2). Epigenetics refers to the heritable phenotypic changes that occur in genes without changing the nucleotide sequence. Studies have reported that a series of cancer-related genes regulated by epigenetic modifications are associated with the occurrence and development of malignant ovarian tumors (7). Epigenetics includes DNA methylation, histone modification, chromosomal remodeling, and non-coding RNA regulation, etc., and affects its function and properties mainly through the regulation of gene transcription or translation. Among them, histone modifications are regulated by histone regulatory enzymes, which can manipulate gene expression (8).

Histones are basic proteins of about 14kDa that are mainly used to regulate gene expression and DNA packaging around nucleosomes. The nucleosome is the functional unit of chromatin, consisting of a histone octamer containing the four core histones H2A, H2B, H3 and H4, which is surrounded by approximately 147 DNA base pairs (bp). Histone linker H1 holds nucleosomes together and is involved in higher-order compression of chromatin. Histones have characteristic tails densely populated with basic lysine and arginine residues. Histone tails are subject to a broad spectrum of covalent posttranslational modifications (PTMs) to control chromatin state, influence chromatin configuration, and recruit protein and enzymatic complexes that regulate gene transcription, replication, and DNA repair process (9, 10). The PTMs of histones often mediate various key biological processes through chromatin modifications that favor the expression or repression of target genes. The ways of histone modification reported by current research include acetylation, methylation, phosphorylation, glycosylation, ubiquitination, ADP-ribosylation and carbonylation modification and so on (11, 12). Among them, the balance of histone acetylation and methylation is regulated by histone acetyltransferase-deacetylase and histone methyltransferase-demethylase, respectively. When this balance is broken, it may lead to abnormal cell function, may even lead to the occurrence and progression of cancer (13).

## Histone acetylation

**Figure 1:**
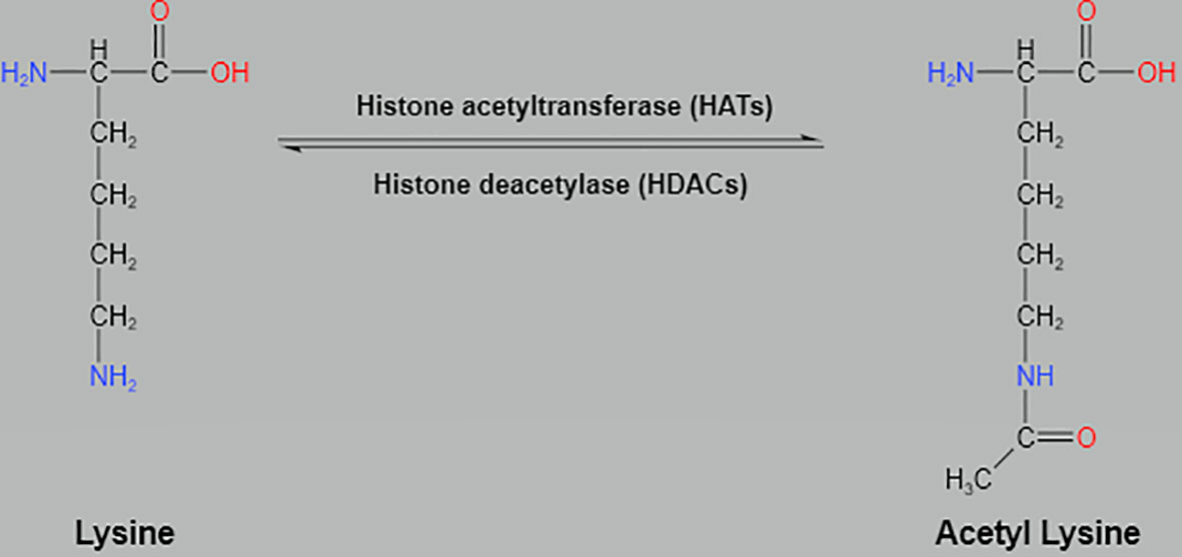
Histone acetylation at the N-terminus lysine by histone acetyltransferases and deacetylation by histone deacetylases.

Histone acetylation is related to the condensed state of chromatin and is a dynamic process regulated by histone acetyltransferase (HAT) and histone deacetylase (HDAC). The addition of acetyl groups can occur at multiple lysine residues on histone tails. HAT weakens electrostatic interactions between negatively charged DNA and histones by neutralizing basic charges on unmodified lysine residues, allowing local chromatin structures to unfold, thereby making achromatin proteins more accessible (10, 14). However, HADC removes negatively charged acetyl groups from histone lysines and restores the positive charge of lysines, resulting in denser chromatin and less accessibility to transcription, thus HADC is associated with chromatin condensed structure and transcriptional repression (**Figure 1**) (4).

## Histone deacetylase

HDAC removes acetyl groups from histone and non-histone proteins, thereby downregulating gene transcription. Deacetylation of positively charged histones keeps them tightly bound to negatively charged DNA, thereby promoting the closure of chromatin structures and preventing gene transcription (15). In addition, HDAC also acts on other molecular mechanisms, including metabolic processes and DNA metabolism, by removing acetyl groups from histones and non-histone proteins. Pathogenic dysregulation of HDAC enzymes has also been proved to play critical roles in DNA replication, genome stability, and DNA damage responses (16).

Mammalian HDACs are divided into four classes based on their structural homology, enzymatic activity, and cellular localization. Class I, II, and IV are zinc-dependent enzymes while class III (also known as sirtuins/SIRTs) are NAD^+^-dependent enzymes. Class I HDACs include HDAC1, 2, 3, and 8, which are expressed in the nucleus. Class II HDACs contain HDAC 4, 5, 6, 7, 9, and 10, which are tissue-specific. Class II consists of two subfamilies: class IIa (HDAC4, 5, 7 and 9), expressed in the nucleus and cytoplasm, and class IIb (HDAC6 and 10), expressed in the cytoplasm. Class III HDACs are mammalian homologues of the yeast silent information regulator (SIR2), include seven members with unique targets (SIRT1-7), are unresponsive to most HADC inhibitors, but require cofactors: NAD^+^. SIRT1, 6, and 7 are present in the nucleus, SIRT3-5 are present in mitochondria, and SIRT2 is mainly present in the cytoplasm (17). Class IV HDAC has only one subtype, HDAC 11, which is expressed in the nucleus and has homology to class I and II HADCs (**Table 1**) (18).

**Table 1:** Classification of HDACs.

Class	Member	Size(aa)	Location
**HDAC I**	HDAC1	483	Nucleus
	HDAC2	488	Nucleus
	HDAC3	428	Nucleus
	HDAC8	377	Nucleus
**HDAC II**	IIa		
	HDAC4	1084	Nucleus/Cytoplasm
	HDAC5	1122	Nucleus/Cytoplasm
	HDAC7	912	Nucleus/Cytoplasm
	HDAC9	1069	Nucleus/Cytoplasm
	IIb		
	HDAC6	1215	Cytoplasm
	HDAC10	669	Cytoplasm
**HDAC III**	SIRT1	747	Nucleus/Cytoplasm
	SIRT2	352	Nucleus
	SIRT3	399	Mitochondria
	SIRT4	314	Mitochondria
	SIRT5	310	Mitochondria
	SIRT6	355	Nucleus
	SIRT7	400	Nucleus
**HDAC IV**	HDAC11	347	Nucleus

Individual HDACs have been reported to be overexpressed in tumors, such as HDAC1 in prostate, gastric, colon, and breast cancer, and HDAC6 in breast cancer, suggesting that aberrant expression of HDACs is largely related to cancer. In turn, siRNA-mediated knockdown of individual HDACs overexpressed in certain tumor cell lines suppresses tumor cell growth and survival (19). Alterations in HDAC expression may therefore play a positive role in tumor onset and progression.

## The role of HDAC in OC

### Class I HADC

Class I HDACs display the strongest histone deacetylase activity, while the remaining classes show a preference for other substrates (20). They localize in the nucleus and can deacetylate all four core histones, regulating genome accessibility for transcription (21). In addition to histones, class I HDACs deacetylate several non-histone proteins, known substrates of HDAC1 are transcription factors such as: p53, E2F1, the corepressor YY1, Proliferating cell nuclear antigen (PCNA), and lysine demethylase 1 (LSD1). The effect of the deacetylation of these proteins are, the destabilization of p53, the inactivation of E2F1, the reduced repressive effect of YY1, the reduced the ability of PCNA to bind to DNA polymerases beta and delta and the enhanced ability of LSD1 to bind histone H3 for its demethylation, respectively (16). Class I HDACs have been reported to be overexpressed in ovarian cancer tissues and play a key role in ovarian carcinogenesis (22). The expression levels of class I HDACs increased with the degree of tumor malignancy and their expression levels were significantly different in various ovarian cancer subtypes, with the highest in mucinous carcinomas (71%), followed by high-grade serous ovarian carcinomas (64%), clear cell carcinoma (54%) and endometrioid carcinoma (36%). HDAC class I expression was generally higher in strongly proliferating tumors, and increased expression of class I HDACs is an independent risk factor for poor prognosis in ovarian malignancies (23). Class I HDACs inhibit the promoter region of RGS2, a regulator of G-protein signaling 2 and an inhibitor of G-protein coupled receptors through accelerating the deactivation of heterotrimeric G-proteins (24). Overexpression of HDAC1 in the nucleus was significantly associated with decreased progression-free survival (PFS) and overall survival (OS) in serous ovarian cancer (25). The expressions of HDAC1 and HDAC2 are positively correlated with the expression of Ki-67 and play an important role in the proliferation of ovarian cancer cells, while the expression of HDAC3 is negatively correlated with the expression of E-Cadherin and played a role in cell adhesion and migration. HDAC1 enhances cell proliferation of OC by promoting cyclinA (26). In the early stage of platinum chemotherapy in ovarian cancer, cellular DNA damage increases HDAC2 expression, which induces chromatin remodeling and reduces the inhibitory effect of platinum on tumor cells (13). HDAC3 activates the PI3K/AKT signaling pathway by combining with human epididymis protein 4 (HE4) to promote the proliferation, invasion and migration of ovarian cancer cells (27). HDAC1 and HDAC7 are overexpressed in ovarian cancer stem cells (CSC) and have the function of maintaining CSC. Cacan E has reported that overexpression of HADC1 causes the down-regulation of G protein signal transduction 2 and 10, and simultaneously inhibits the inhibitory effect of platinum on tumor cells (28, 29). Kim believes that overexpression of HDAC1-4 is associated with the development of platinum resistance in OC (30).

### Class II HADC

Class IIa HDACs can shuttle between the nucleus and the cytoplasm, and have a dual nature of scaffold proteins and active enzymes (16). Class IIb HDACs are primarily localized to the cytoplasm, and thus the primary physiological targets are non-histone proteins. HDAC6 can stabilize microtubules by deacetylating α-tubulin and can control p53 activity by targeting acetyl-Lysine 381/382. Another target of HDAC6 deacetylation activity is MutS homologue-2 (MSH2), a protein involved in DNA mismatch repair (16). HDAC10 exhibits strong polyamine deacetylase activity but poor lysine-deacetylase activity compared to HDAC6 (31). HDAC10 may have deacetylation activity on cytoplasmic proteins such as heat shock cognate protein 70 (Hsc70)/heat shock protein (Hsp70). In addition, HDAC10 can also target MSH2 through deacetylation-specific lysines to enhance MSH2 activity *in vitro (* 16). HDAC4 may mediate the interaction between p53 and RAS signaling to actively control ovarian cancer cisplatin resistance through dysregulation of apoptosis and autophagy (32). HDAC6 is overexpressed in OC, and HDAC6 upregulation is thought to be associated with poor prognosis and poor chemoresistance in patients with advanced high-grade serous ovarian cancer (33). However, Ali et al. believe that the overexpression of HDAC6 in OC is a favorable prognostic marker (34). HDAC6 is thought to play a vital role in oncogene Ras-induced transformation, is a major factor in tumorigenesis and maintenance of the transformed phenotype, and is involved in cancer metastasis and migration. HDAC6 is required for activation of MAPK and PI3K signaling cascades, which may contribute to anchorage-independent growth of cancer cells. HDAC6 may contribute to tumorigenesis, at least in part, by promoting activation of Ras and downstream PI3K and MAPK pathways (35). HADC6 gene is also an estrogen-regulated gene and a potential indicator of prognosis in estrogen-dependent tumors such as breast cancer (36). Serous ovarian cancer patients with high HDAC9 expression have a poor prognosis, while non-serous ovarian cancer patients with high HDAC9 expression have a higher survival rate. In serous ovarian cancer, overexpressed HDAC9 may activate epithelial–mesenchymal transition (EMT) by increasing nuclear accumulation of FOXO1. In non-serous ovarian cancer, HDAC9 may inhibit cell migration by inhibiting β-catenin signaling (37). Low HDAC10 levels correlate with platinum sensitivity of tumors, and inhibition of HDAC10 enhances the efficacy of platinum chemotherapy in primary ovarian cancer cells (38).

### Class III HADC

SIR2 gene was first discovered in budding yeast and described as a regulator of chromatin structure. SIR2 homologues, termed “sirtuins (SIRTs)”, were subsequently identified in bacteria, plants, and mammals as a family of highly conserved proteins (39), belonging to class III HADCs. Mammalian SIRT family members have distinct biological functions due to differences in subcellular localization, expression patterns, and substrate binding (40). In addition to being involved in stress resistance, genome stability, energy metabolism, and longevity, evidence suggests that SIRT family members are involved in tumorigenesis and development (17, 41). SIRT1 is one of the most studied members, regulating DNA repair and metabolism, tumorigenesis, cellular senescence, mammalian cell survival under stress, cell cycle progression and processes such as apoptosis by deacetylating histone and non-histone substrates such as p53 (42, 43). SIRT1 is the hub gene and pathogenic gene of OC. The function of SIRT1 in EOC shows marked heterogeneity (44). SIRT1 expression is elevated in endometrioid, mucinous, and clear cell ovarian cancer, but not in serous ovarian cancer. Meanwhile, elevated SIRT1 expression is an important predictor of shortened survival (45). Furthermore, SIRT1 regulates cisplatin sensitivity in OC through BRCA1-SIRT1-EGFR signaling, and SIRT1 level is elevated in cisplatin-resistant OC (46). Overexpression of SIRT1 plays a key role in chemoresistance in ovarian malignancies and serves as a predictor of poor clinical prognosis (47). The expression of SIRT2 is decreased in serous ovarian cancer, and down-regulation of SIRT2 promotes the expression of cyclin-dependent kinase 4 (CDK4), thereby promoting the proliferation, migration and invasion of serous ovarian cancer cells *in vitro (* 48). SIRT2 expression is elevated in cisplatin-sensitive ovarian cancer cells and is associated with cisplatin-induced ROS. Overexpression of SIRT2 enhances the sensitivity of non-resistant cells to cisplatin (49). The expression of SIRT3 is significantly downregulated in metastatic ovarian cancer (50), but low SIRT3 expression is not associated with a reduction in OS in patients (51). SIRT3 can regulate ovarian cancer cell autophagy through multiple distinct molecular signaling pathways, including p62, 5’AMP-activated protein kinase, and mitochondrial ROS superoxide dismutase pathways (52). Additionally, SIRT3 mediates the antitumor effects of mitochondrial complex I inhibitors in human ovarian cancer cells by inducing energy stress and apoptosis (53). SIRT3 also regulates the sensitivity of ovarian cancer cells to cisplatin by regulating the mitochondrial fission process (54). SIRT4 and SIRT6 are novel prognostic biomarkers with competing functions in serous ovarian cancer. SIRT4 is associated with immune responses during oocyte maturation, and SIRT6 is involved in immune-related disease pathways and mitochondrial metabolism-mediated DNA translation (55). SIRT5 is overexpressed in ovarian cancer tissues and promotes cisplatin resistance in ovarian cancer cells by inhibiting cisplatin-induced ROS-dependent DNA damage by regulating the nuclear factor erythroid 2-related factor 2 (Nrf2)/heme oxygenase 1 (HO-1) pathway (56). Zhang et al. have reported that SIRT6 is downregulated as a tumor suppressor in OC. SIRT6 inhibits ovarian cancer cell proliferation through Notch3 downregulation and correlates with ovarian cancer prognosis (57)。However, Bandopadhyay et al. believe that SIRT6 is overexpressed in ovarian cancer tissues. Increasing SIRT6 expression promotes the invasiveness of ovarian cancer cells but does not alter cellular proliferation. SIRT6 promotes mitochondrial fission and subsequent cellular invasion in ovarian cancer (58). SIRT7 is overexpressed in OC, and it may inhibit ovarian cancer cells by inducing changes in apoptosis-related molecules and NF-κB family subunits (59). He et al. have found that overexpression of SIRT3/5/6/7 is associated with good prognosis in ovarian cancer patients, while elevated levels of SIRT1/4 are associated with poorer patient survival (60).

### Class IV HADC

HDAC11 is responsible for the deacetylation of core histones and is a key factor in the regulation of transcription and the cell cycle (61). The association of HDAC11 OC has been rarely reported. Deubzer et al. have reported that deletion of HDAC11 inhibits the metabolic activity of ovarian cancer cells and leads to cell death (62).

## Application of HDACIs in OC

It has been widely recognized in recent years that HDACs are promising targets for therapeutic interventions intended to reverse aberrant epigenetic associated with cancer. Therefore, there has been considerable effort to develop HDAC inhibitors (HDACIs) (19). Epigenetic therapy is administered alone or in combination with standard therapy in clinical trials to resensitize tumors or prevent resistance to therapy (15). HDACIs can be divided into four groups including aliphatic fatty acids, hydroximic acids, benzamides, and cyclic peptides. HDACIs inhibit deacetylation of lysines in histone and non-histone cellular proteins, induce hyperacetylation and open chromatin conformation. HDACIs can reverse the transcriptional repression of tumor suppressor genes, promote anti-tumor environment, induce tumor cell growth arrest, apoptosis and inhibit tumor angiogenesis to exert anti-cancer activity (15). Besides, HDACIs exert multiple mechanisms of action such as activation of the apoptotic pathway, cell cycle arrest, apoptotic induction occurs *via* extrinsic (death receptor) or intrinsic (mitochondrial) pathways, both of which lead to caspase activation and cell death induction because HDAC have many cellular and target proteins (24). HDACIs have been shown to be successful in treating hematological malignancies such as multiple myeloma, acute lymphoblastic leukemia and acute myeloid leukemia (63). To date, the United States Food and Drug Administration (FDA) has approved four HDACIs: vorinostat (MK0683), romidepsin (FK288), belinostat (PDX101), panobinostat (LBH589) for clinical cancer treatment. Among them, romidepsin is a class I HDAC1/2-selective inhibitor, and the other three are pan-HDACIs (63, 64). Epigenomic alterations have emerged as attractive prognostic markers and drug therapy targets to improve outcomes in patients with OC, especially platinum-resistant and recurrent OC (2).

Preclinical studies have shown the anti-tumor activity of HDACIs in ovarian cancer. *In vitro* studies have suggested that HDACIs sensitize ovarian cancer cells to DNA-damaging drugs such as platinum by increasing apoptosis-mediated cell death induced by platinum-based chemotherapy (25). Romidepsin causes DNA damage-induced apoptosis and enhances the antitumor effects of cisplatin both *in vitro* and *in vivo (* 65). Panobinostat inhibits cellular growth and improves cisplatin-resistance in ovarian cancer cells (64, 66, 67). Researchers are also exploring the combined therapy of HDACIs and other anti-tumor drugs. Panobinostat enhances olaparib efficacy by modifying expression of homologous recombination repair and immune transcripts in ovarian cancer (68). Panobinostat synergizes with chloroquine cause induce DNA damage and inhibit DNA repair (69). Priming with panobinostat sensitizes ovarian cancer cells to treatment with cisplatin and heat shock protein 90 (HSP90) inhibitors (70). Valproic acid enhances the anti-cancer activity of the PARP1 inhibitor niraparib in ovarian cancer cells (71). Suberoylanilide hydroxamic acid (SAHA) inhibits the growth of paclitaxel-resistant ovarian cancer cells and reduces migration by the induction of cell-cycle arrest, apoptosis and autophagy (72).

Currently many HDACIs are under clinical trials in patients suffering from ovarian cancer (**Table 2**). Valproic acid (VPA) belongs to aliphatic fatty acid family and targets Class I and IIa of HDACs. In a phase II study of hydralazine and VPA treating refractory solid tumors, patients received hydralazine at 182 mg for rapid, or 83 mg for slow, acetylators, and VPA at 40 mg/kg, beginning a week before chemotherapy. As a result, 17 patients were evaluable for toxicity and 15 for response. Primary sites included cervix (3), breast (3), lung (1), testis (1), and ovarian (6) carcinomas. A clinical benefit was observed in 12 (80%) patients: four partial response (PR), and eight stable disease (SD). The most significant toxicity was hematologic. Reduction in global DNA methylation, histone deacetylase activity, and promoter demethylation were observed. The data suggested the clinical benefit noted with hydralazine and VPA in this selected patient population (73). The research group is planning to carry out a phase III clinical trial (ClinicalTrials.gov Identifier: NCT00533299). Nakka et al (74) conducted a prospective, single-arm, open-label phase II study of VPA combined with etoposide in platinum-resistant ovarian cancer (PROC) patients. Patients received oral VPA 60 mg/kg/day in three divided doses for 3 days (D1-D3), followed by oral etoposide 50 mg once daily for two consecutive weeks (D4-D17). The primary endpoint was the overall response rate (ORR). The secondary endpoints were PFS, OS, and toxicity. 27 patients were enrolled, and 18 were evaluable for the response after 4 months. 9 patients were lost from follow-up before achieving the primary endpoint. The median number of prior lines of treatment was 2 (1–3). ORR was 0% according to GCIG criteria. The disease was stable in 2 patients [clinical benefit rate (CBR) of 11%]. The median OS and PFS were 7 months and 2 months, respectively. Grade ≥ 3 adverse events were reported in 6 (33%) patients. The addition of VPA to oral etoposide in patients with PROC and poor general condition was not helpful and failed to improve responses compared to those historically achieved with single-agent etoposide.

**Table 2:** Clinical trials of HDACIs treating ovarian cancer.

Classification	Drug	Targeted HDACs	Combination	Conditions	Phase	Reference
**Aliphatic fatty acids**	Valproic acid (VPA)	Class I and IIa	Topotecan, Hydralazine	Refractory OC	Phase II, III	(73), NCT00533299
			Azacitidine, Carboplatin	OC	Phase I	NCT00529022
			Dexamethasone	OC	Phase I	NCT02520115
			Etoposide	Platinum-resistant OC	Phase II	(74)
**Hydroximic acid**	Panobinostat (LBH589)	Class I and II	Gemcitabine	Advanced OC	Phase I	(75)
	Belinostat (PDX101)	Class I and II	5-Fluorouracil (5-FU)	Advanced OC	Phase I	NCT00413322
			–	Advanced EOC, ovarian low malignant potential tumors	Phase II	NCT00301756
			Carboplatin	Recurrent or persistent platinum-resistant OC	Phase II	(76)
			Carboplatin, Paclitaxel	OC in need of relapse	Phase II	NCT00421889
			–	Advanced OC	Phase I	NCT00413075
			Carboplatin, Paclitaxel	Recurrent EOC	Phase Ib/II	(77)
			Ribociclib	Recurrent OC	Phase I	NCT04315233
			Carboplatin, Paclitaxel	Advanced, refractory OC	Phase I	(78)
			–	Platinum- resistant EOC, micropapillary ovarian tumors	Phase II	(79)
			Talazoparib	Metastatic OC	Phase I	NCT04703920
	Vorinostat (MK0683)	Class I and II	Carboplatin,Gemcitabine	Recurrent, platinum-sensitive OC	Phase Ib/II	NCT00910000
			Paclitaxel, Carboplatin	Advanced OC	Phase I/II	NCT00976183
			–	Recurrent or persistent EOC	Phase II	NCT00132067
			Paclitaxel, Carboplatin	Recurrent EOC	Phase I	NCT01249443
			–	Advanced OC	Phase I	NCT00045006
			Carboplatin, Gemcitabine	Recurrent, platinum-sensitive EOC	Phase I	(80)
			–	Persistent or recurrent EOC	Phase II	(81)
			Paclitaxel, Carboplatin	Recurrent OC	Phase I/II	NCT00772798
	Quisinostat	Class I (82)	Paclitaxel+Carboplatin or Gemcitabine +Cisplatin	EOC	Phase I	NCT02728492
			Paclitaxel, Carboplatin	Advanced EOC	Phase II	NCT02948075
	Entinostat (MS275)	HDAC 1/2	Avelumab	Advanced EOC	Phase Ib/II	NCT02915523
			–	Advanced OC	Phase I	NCT00020579
			Olaparib	Recurrent, platinum-refractory, resistant OC	Phase I	NCT03924245
	Ricolinostat	HDAC6	Paclitaxel	Recurrent OC	Phase Ib	(83)
**Cyclic peptides**	Romidepsi (FK288)	Class I	–	Recurrent EOC	Phase II	NCT00091195
			–	Relapsed or refractory advanced EOC	Phase II	NCT00085527

Hydroximic acid seems to be the largest subgroup of HDACIs, including vorinostat, belinostat, panobinostat and newly-developed HDACIs. A phase I clinical trial was conducted for treating advanced solid tumors with pabinostat and gemcitabine to evaluate the safety and tolerability. Patients received oral panobinostat administered 2 or 3 times weekly (continuous or intermittent dosing in combination with intravenous gemcitabine administered on days 1, 8, and 15 every 28 days or on days 1 and 8 every 21 days). Finally, a total of 63 cycles of study treatment were administered to 17 patients over 5 different dose levels. Dose-limiting toxicities occurred at all dose levels. In all instances, dose-limiting toxicities were due to grade 4 myelosuppression or myelosuppression warranting dose modifications during the first treatment cycle. Nonhematologic toxicities were mild to moderate in intensity and consisted of anorexia, constipation, diarrhea, fatigue, nausea, vomiting, and rash. One patient with OC had an unconfirmed partial response, and 5 patients had stable disease lasting more than 4 cycles. The result suggested that dosing of the combination regimen of panobinostat and gemcitabine is limited by myelosuppression (75). In another phase I trial of belinostat in combination with carboplatin and/or paclitaxel in 23 patients with solid tumors, cohorts of three to 6 patients were treated with escalating doses of belinostat administered intravenously once daily, days 1-5 q21 days; on day 3, carboplatin (area under the curve (AUC) 5) and/or paclitaxel (175mg/m^2^) were administered 2-3h after the end of the belinostat infusion. Results revealed that all 23 patients received 600-1000 mg/m^2^ per day of belinostat with carboplatin and/or paclitaxel. No dose-limiting toxicity was observed. The maximal administered dose of belinostat was 1000 mg/m^2^ per day for days 1-5, with paclitaxel (175mg/m^2^) and carboplatin AUC5 administered on day 3. Grade III/IV adverse events were (n; %): leucopenia (5; 22%), neutropenia (7; 30%), thrombocytopenia (3; 13%) anemia (1; 4%), peripheral sensory neuropathy (2; 9%), fatigue (1; 4%), vomiting (1; 4%) and myalgia (1; 4%). The pharmacokinetics of belinostat, paclitaxel and carboplatin were unaltered by the concurrent administration. There were two PRs (one rectal cancer and one pancreatic cancer). A third patient (mixed mullerian tumor of ovarian origin) showed a complete CA125 response. In addition, six patients showed a stable disease lasting > or =6 months. The data suggested the combination was well tolerated, with no evidence of pharmacokinetic interaction (78). A phase Ib/II study was performed by Dizon et al. to further evaluate the clinical activity of belinostat, carboplatin, and paclitaxel (BelCaP), with an exploratory phase 2 expansion planned specifically for women with recurrent EOC. Thirty-five women were treated on the phase 2 expansion cohort. BelCap was given as follows: belinostat, 1000 mg/m² daily for 5 days with carboplatin, AUC5; and paclitaxel, 175 mg/m² given on day 3 of a 21-day cycle. The primary end point was ORR. The results showed that 54% had received more than two prior platinum-based combinations, 16 patients (46%) had primary platinum-resistant disease, whereas 19 patients (54%) recurred within 6 months of their most recent platinum treatment. The median number of cycles of BelCaP administered was 6 (range, 1-23). Three patients had a complete response (CR), and 12 had a PR, for an ORR of 43% (95% confidence interval, 26%-61%). When stratified by primary platinum status, the ORR was 44% among resistant patients and 63% among sensitive patients. The most common drug-related adverse events related to BelCaP were nausea (83%), fatigue (74%), vomiting (63%), alopecia (57%), and diarrhea (37%). With a median follow-up of 4 months (range, 0-23.3 months), 6-month PFS is 48% (95% confidence interval, 31%-66%). Median OS was not reached during study follow-up, suggesting the combination was reasonably well tolerated and demonstrated clinical benefit in heavily-pretreated patients with EOC (77). A phase II study was performed to evaluate the activity of belinostat in women with metastatic or recurrent platinum resistant EOC and micropapillary LMP ovarian tumors, both groups had received no more than 3 prior lines of chemotherapy. Belinostat 1000 mg/m^2^/d was administered iv days 1-5 of a 21d cycle. The results revealed that eighteen patients with EOC and 14 patients with LMP tumors were enrolled on study. Belinostat was well tolerated with no grade four toxicity (179 cycles). Grade 3 toxicity consisted of thrombosis (3 patients), hypersensitivity (1) and elevated ALP (1). One patient with LMP tumor had a PR (unconfirmed) and 10 had SD, 3 were non-evaluable. Median PFS was 13.4 months (95% CI, 5.6-not reached). Best response in patients with EOC was SD (9 patients) and median PFS was 2.3 months (95% CI, 1.2-5.7 months). Belinostat is well tolerated in both patient groups and shows some activity in patients with LMP disease (79). Gynecologic Oncology Group (GOG) conducted a phase II clinical trial to evaluate the effect of belinostat in combination with carboplatin in women with platinum-resistant ovarian cancer. Belinostat was dosed at 1,000 mg/m^2^ daily for five days with carboplatin AUC5 on day three of 21-day cycles. The primary endpoint was ORR, using a two-stage design. 29 women enrolled on study and 27 were evaluable. The median number of cycles given was two (range 1–10). One patient had a CR and one had a PR, for an ORR of 7.4% (95% CI, 0.9% - 24.3%). Twelve patients had SD while eight had increasing disease. Response could not be assessed in five (18.5%). Grade 3 and 4 events occurring in more than 10% of treated patients were uncommon and limited to neutropenia (22.2%), thrombocytopenia (14.8%), and vomiting (11.1%). The median PFS was 3.3 months and OS was 13.7 months. PFS of at least six months was noted in 29.6% of patients. Due to the lack of drug activity, the study was closed after the first-stage (76). A phase I study was performed by Matulonis et. al, treating fifteen recurrent and platinum-sensitive EOC patients with vorinostat, carboplatin and gemcitabine to determine the maximally tolerated dose of this combination. Doses of carboplatin and gemcitabine were AUC4 on day 1 and 1000 mg/m^2^ on days 1 and 8, respectively; cycles were administered every 21 days. The first dose level (DLA) tested vorinostat as daily oral dosing from days 1 to 14. DLB tested twice daily (BID) vorinostat dosing on days 1-3 and 8-10. DLC tested BID vorinostat dosing on days 1, 2, 8, and 9, starting vorinostat 1 day prior to initiation of carboplatin and gemcitabine, and DLD tested vorinostat on days 1 and 2 with chemotherapy starting on day 2. Results showed all four DLs tested resulted in dose-limiting toxicities, and no MTD was determined. Toxicities were mostly hematologic. Seven patients were evaluable for RECIST assessment, and six of them had PR *via* RECIST. The conclusion was drawn that combination of carboplatin, gemcitabine and vorinostat has activity in relapsed platinum-sensitive ovarian cancer, but was difficult to combine because of hematologic toxicities. No maximally tolerated dose was found, and the study was terminated early (80). GOG conducted a phase II trial to assess the activity and toxicity of vorinostat in patients with recurrent or persistent EOC or primary peritoneal carcinoma. Patients were treated with a 400 mg daily oral dose of vorinostat and continued on treatment until disease progression or unacceptable toxicity. The primary endpoints were PFS at 6 months and toxicity. Secondary endpoints were tumor response, duration of PFS and duration of OS. Twenty-seven women were enrolled. Two women survived progression-free over 6 months, with one having a PR. Two grade 4 toxicities were reported (one leukopenia and one neutropenia). The most common grade 3 toxicities were constitutional (3/27; 11%) and gastrointestinal (3/27, 11%). Other grade 3 toxicities included neutropenia, metabolic abnormalities, and thrombocytopenia (two patients each, 7%) as well as neurologic complaints and pain (1 patient each; 4%). It was concluded that vorinostat is well tolerated but had minimal activity as a single agent in unscreened patients with recurrent platinum-refractory ovarian or primary peritoneal carcinoma (81).

Results of clinical trials above suggest the majority of combination therapy show promising outcomes while the combination of panobinostat and gemcitabine in advanced OC is limited by myelosuppression. And the combination of carboplatin, gemcitabine plus vorinostat has activity in relapsed platinum-sensitive ovarian cancer, but also displays hematologic toxicities and ends up in termination (75, 80). Obviously, a huge issue concerning the development of HDACIs in the therapy of ovarian cancer is the severe side effects due to cytotoxicity to normal cells. Besides, it is reported that selective HDAC inhibition is superior to pan-HDAC inhibition in modulating cisplatin potency in ovarian cancer (84). Therefore, better selective inhibitors of HDAC with higher tumor efficacy and lower toxic side effects should be explored for ovarian cancer treatment. To date some new HDACIs have already been developed and are being tested. Bai et al. have discovered a novel HDACI structure, Z31216525 targeting HDAC7, that inhibits the proliferation of OC cells (85). A novel HDAC6-selective inhibitor, A452 has been found to enhance anticancer effects of paclitaxel in OC cells (86). A new SIRT1 inhibitor, MHY2245, induces autophagy and inhibits energy metabolism *via* PKM2/mTOR pathway in OC cells (87). Moreover, with development of resistance to classical chemotherapy and the emerging immunotherapy, a combinatory treatment with HDACIs might be a new approach to fight against this deadly disease. Studies of HDACs are more nascent, yet clinical trials in inhibitors of these proteins are underway.

## Conclusion

In this review, we summarize the latest studies on the classification and mechanisms of action of HDACs and the clinical application of their inhibitors in ovarian cancer. Some HDACIs are currently in clinical trials for different types of ovarian cancers alone or in combination with DNA demethylating agents, chemopreventive drugs, or classical chemotherapy drugs. Epigenetic therapies, especially modulators of histone-modifying enzymes, have become a hot topic in ovarian cancer research. HDAC inhibitor has also been shown to be a promising candidate for cancer therapy, but its relatively low specificity may lead to adverse side effects in ovarian cancer patients, hindering its broad clinical application. Based on this, scientists are working to develop a new generation of HDACIs and explore combination therapy with maximum antitumor activity and minimum side effects.

## Author contributions

FG contributed in the generation of concept, literature search, literature review and draft of the manuscript. HW contributed significantly in the literature review and the revision of the manuscript. All authors of this manuscript have read and approved the manuscript for submission.

## Funding

The authors greatly appreciate the financial support from Clinical Research Foundation of West China Second Hospital (grant #KL113), and Sichuan Province Science and Technology Program (grant #2021YFS0126).

## Conflict of interest

The authors declare that the research was conducted in the absence of any commercial or financial relationships that could be construed as a potential conflict of interest.

## Publisher’s note

All claims expressed in this article are solely those of the authors and do not necessarily represent those of their affiliated organizations, or those of the publisher, the editors and the reviewers. Any product that may be evaluated in this article, or claim that may be made by its manufacturer, is not guaranteed or endorsed by the publisher.
